# Cytotoxic effects on cancerous and non-cancerous cells of *trans*-cinnamaldehyde, carvacrol, and eugenol

**DOI:** 10.1038/s41598-021-95394-9

**Published:** 2021-08-11

**Authors:** Saurav Ranjitkar, Delong Zhang, Fei Sun, Saleh Salman, Wu He, Kumar Venkitanarayanan, Edan R. Tulman, Xiuchun Tian

**Affiliations:** 1 Departments of Animal Science, University of Connecticut, 1390 Storrs Road, Storrs, CT 06269 USA; 2 Flow Cytometry Facility, Center for Open Research Resources and Equipment, Storrs, CT USA; 3 Departments of Pathobiology, University of Connecticut, Storrs, CT USA

**Keywords:** Biotechnology, Cell biology, Molecular biology, Oncology

## Abstract

Essential oils and their active components, referred here as plant derived antimicrobials (PDAs), have been used for their antimicrobial, anti-inflammatory and antioxidant properties. Many reports also document PDAs’ cytotoxic effects on cancerous cells, raising the hope that they could be used for cancer treatments. Due to the lack of specificity, we hypothesize that PDAs are cytotoxic to both cancerous and non-cancerous cells. *Trans*-cinnamaldehyde (TCA), carvacrol, and eugenol were assessed for their cytotoxicity on cancerous HeLa cells and normal skin fibroblasts (CCD-1123Sk, CCD) by MTT and LDH assays, flow cytometry, and reverse transcription quantitative PCR (RT-qPCR). After 24 h of treatment, carvacrol and TCA significantly decreased cell viability (by more than 50%) at 100 µg/ml, whereas eugenol was ineffective up to 400 µg/ml. Cell detachment and significantly increased apoptosis were observed with 100 µg/ml of TCA on both cell types. RT-qPCR for apoptotic genes (*BCL2, CASP3 and CASP8*) and necrosis genes (*MLKL, RIPK1 and RIPK3*) did not show significant differences between control and treated cells of both types, with the exception of eugenol-treated HeLa cells in which expression of *BCL2, MLKL* and *RIPK1* was significantly higher than controls. Taken together, we conclude that the three PDAs studied here exhibited similar cytotoxic effects on both cancerous and non-cancerous cells.

Essential oils have been used for centuries around the world in foods, cosmetics and as antimicrobials and pharmaceuticals to prevent and treat diseases^1^. The bioactive constituents of essential oils are secondary plant metabolites and their derivatives^2^. An active ingredient of cinnamon oil, for example, is cinnamaldehyde, which predominately occurs in the trans form, *trans*-cinnamaldehyde (TCA)^3^. Similarly, eugenol (EU) and carvacrol (CAR) are the main active ingredients of the essential oils from oregano (*Origanum vulgare*) and clove (*Syzgium aromaticum*)^4^, respectively. These plant compounds, referred together here as plant-derived antimicrobials (PDAs), have been found to possess a plethora of biological activities including anti-microbial, anti-inflammatory, anti-oxidant, and anti-cancerous effects^5,6^. The antimicrobial and anti-cancerous effects are exerted through cell membrane damages, free radical generation, reduced energy metabolism, apoptosis, cell cycle arrest and inhibition of other cellular pathways such as the MAPK^7–9^.

The anti-cancerous activity of PDAs has been extensively tested in many cancer cell lines such as HeLa and HCT15 cells^10–13^. Using the colorimetric assay of MTT (3-(4,5-dimethylthiazol-2-yl)-2,5-diphenyltetrazolium bromide), TCA has been reported to exhibit time- and dose-dependent inhibition on the metabolic activities of HeLa cells^14,15^. TCA appeared to lead to apoptosis as shown by activated caspase cascade, DNA fragmentation, and loss of mitochondrial potentials in various tumor cell lines such as HCT15, SK-MEL-2, and K562 cells^16,17^. Carvacrol exhibited effects comparable to TCA on cancer cell lines^18,19^ and additionally arrested human prostate cancer cells at G0/G1^20^. This anti-proliferative effect of carvacrol was also seen in primary rat neurons and N2a neuroblastoma cells^21^. Likewise, eugenol acted in HeLa cells as an anti-proliferative and cyto-lytic agent in a dose-dependent manner as shown by MTT and LDH (Lactate Dehydrogenase assay), respectively^22,23^. Moreover, eugenol also appears to be anti-metastatic by downregulating genes such as *MMP2* and *MMP9*, which are correlated to metastasis^24^.

Despite extensive reports that PDAs are anti-cancerous in cell cultures, virtually all studies lacked non-cancerous cell lines as the control. These results gave the illusion that PDAs may have specific cytotoxic effects on cancerous cells only and not on non-cancerous cells. We hypothesize conversely that, due to common mechanisms of cytotoxicity and the lack of a specific targeting pathway, PDAs should have the same effects on non-cancerous cells as on cancerous cells. Here we directly compared cytotoxicity of three PDAs (TCA, carvacrol and eugenol) on cancerous cells (HeLa) and non-cancerous fibroblast cells (CCD-1123Sk; CCD) to support our hypothesis.

Fibroblasts are the most common cells used in cell culture experiments and are easy to culture. Also, fibroblast cells are present in every tissue of the body (except for the blood). Therefore, if fibroblasts are negatively affected by PDAs, the entire body would be affected if PDAs were to be used as cancer treatments. Therefore, we chose a “universal” cell type in our study to determine the broadest effects of PDAs on non-targeted cells. HeLa cells were chosen as they are the most commonly used cancer cell line for anti-cancerous studies.

## Materials and methods

### Chemicals and reagents

All chemicals and reagents were purchased from Sigma (St Louis, MO) unless noted otherwise. 10% stock solutions of TCA (C80687-25G, Lot # MKCD4749), carvacrol (W224502-100G-K, Lot # MKBW8250V), and eugenol (E51791-100G, Lot # STBG9481) were made using dimethyl sulfoxide (DMSO) as the vehicle and stored at 4 °C. Working concentrations of each PDA, ranging from 0–800 µg/ml, were made by diluting the stock solution with Iscove's Modified Dulbecco's Medium (IMDM) on the day of usage. The highest concentration of DMSO did not exceed 0.72% which was also used for controls.

### Cell culture

CCD-1123Sk (CCD) and HeLa S3 cells (ATCC CCL-2.2) were obtained from ATCC (Manassas, VA). Both types of cells were maintained in IMDM with 10% fetal bovine serum (FBS, Thermo Fisher Scientific, Hampton, NH) and L-glutamine (2 mM, GlutaMAX, Gibco) in 5% CO_2_ in humidified air at 37 °C. The same medium was used for all experiments, and antibiotics were not used. For every experiment, the same passage number for the two cell types were used and cells were not used after the 7^th^ passage from receipt of the cell lines.

### MTT assay for metabolic activity

The MMT assay is a sensitive and reliable indicator of the cellular metabolic activity^25^. The Vybrant MTT Cell Proliferation Assay Kit (Cat No: V13154, Thermo Fisher, Waltham, MA) was purchased and used by following the procedure of the manufacturer. HeLa and CCD cells (10^4^ cells/200 µl/well) were seeded in 96-well plates and cultured for 24 h for proper attachment. Subsequently, the medium was replaced with that containing working concentrations of each PDA. The final concentrations of TCA, EU and CAR were 0, 12.5, 25, 50, 100, 200, 400 and 800 µg/ml. Two technical replicates were included for each treatment, and the experiment was conducted 3 times. PDA treatment was removed after 24 h by replacing PDA medium with 100 µl of fresh medium. Then 10 µl of 12 mM of MTT solution was added to each well, mixed, and incubated at 37 °C in 5% CO_2_. After 4 h, 75 µl of medium was removed and 50 µl of 100% DMSO added per well and thoroughly mixed to dissolve the purple formazan crystals. Plates were incubated for 10 min at 37 °C, wells subjected to additional mixing and removal of air bubbles, and absorbance was read at 540 nm using a plate reader (BioTek Synergy 2).

### LDH assay for cytotoxicity

The CyQUANT LDH Cytotoxicity Assay (Thermo Fisher, Cat No: C20301) was purchased to determine the release of lactate dehydrogenase (LDH) into the medium, a measure of cell death/plasma membrane damage. Seeding and treatment of cells were similar to the MTT assay, with the exception of cell number (5 × 10^3^ cells/well) for the optimal functioning of the assay. First, the spontaneous and maximum LDH activity were set up as follows: 24 h after cell seeding, 10 µl of sterile water (spontaneous LDH control) or lysis buffer (maximum LDH control) were added to cells not treated with PDAs. The reactions were mixed and incubated for 45 min at 37 °C in 5% CO_2_. Subsequently and from all sample wells (spontaneous and maximum LDH controls and PDA-treated tests), 50 µl of culture medium were transferred to a new 96-well plate. To this 50 µl of LDH reaction mixture was added, mixed, and incubated for 30 min at room temperature. To terminate the reactions, 50 µl of stop solution were added, mixed, and bubbles removed by centrifugation. Absorbance of the reaction mixtures was taken at 490 nm and 680 nm. To determine LDH activity, the 680 nm absorbance value was subtracted from that of the 490 nm. Then cytotoxicity was calculated using the formula:

$$ Cytotoxicity \left( \% \right) = \left( {\frac{compound\, treated\, LDH\, activity - Spontaneous\, LDH\, activity }{{Maximum\, LDH\, activity - Spontaneous\, LDH\, activity }}} \right)*100\% $$

### Apoptotic assay and cell morphology

Mitochondrial Membrane Potential Apoptosis Kit was purchased from Thermo Fisher (Cat No. V35116). For flow cytometry, 5 × 10^5^ cells per well were plated in 6-well plates and incubated for 24 h at 37 °C in 5% CO_2_. The medium was then replaced with 2 ml IMDM containing 100 µg/ml of TCA—a level chosen because it induced cytotoxicity in prior experiments. Cells were incubated with the TCA treatment for 4 h, then harvested by trypsinization, centrifugation, and re-suspension in 1 ml of IMDM. To stain live cells, 4 μl of 10 μM MitoTracker Red working solution were added and incubated at 37 °C in 5% CO_2_ for 30 min. Cells were then washed with phosphate-buffered saline to remove the red stain. To stain apoptotic cells, 5 μl of Annexin V dye was added to cells suspended in 1X Annexin binding buffer and incubated for 5 min at room temperature. Next, 400 μl of 1X Annexin binding buffer was added, mixed gently, and placed on ice. Cells were analyzed by flow cytometry (BD LSRFortessa X-20) at 530 and 585 nm.

### RNA isolation and real-time quantitative PCR (RT-qPCR)

To determine if the apoptosis and necrosis pathways were involved in the effects of PDAs, we opted to compare, between treated and non-treated cells, gene expression for *BCL2, CASP3*, and *CASP8*, as they are classical genes in the apoptotic pathway. Similarly, *MLKL, RIPK1, and RIPK3* were selected to investigate effects on the necrosis pathway^26–28^. RNA was isolated from the frozen samples using RNeasy Mini Kit. For TCA and CAR treatment, samples were collected from control and 100 µg/ml-treated wells, whereas for eugenol treatment, samples were collected from control and 400 µg/ml-treated wells. For cDNA synthesis, a BioRad iScript cDNA Synthesis Kit was used. Briefly, 5× iScript reaction mix (4 µl), iScript reverse transcriptase (1 µl of 200 U/µl), RNA template (50 ng), and nuclease-water was mixed to a total reaction volume of 20 µl. The cDNA synthesis was conducted in a PCR thermocycler (MJ Research 200) at 25 °C for 5 min, 46 °C for 20 min, and inactivation at 95 °C for 1 min. After cDNA synthesis, two rounds of PCR were performed. Due to low mRNA yield under the treatment regimen, we performed a linear pre-amplification PCR to obtain sufficient cDNA for the Real-Time qPCR. The first round of PCR was 12 cycles of amplification (MJ Research 200) in 96 well plate with respective primers using Dream Taq PCR reagents (Thermo Fisher, Waltham, MA). The reaction setup was 10× Dream Taq buffer (2 µl), forward and reverse primers (0.5 µl each of 10 µM), dNTP (2 µl of 2 mM), Polymerase (0.5 µl of 5 U/µl), cDNA in 2 µl, and 13 µl of water. The temperature profile for the reaction was: 95 °C for 1 min, 60 °C for 30 s, and 65 °C for 5 s (for 12 cycles), and finally 4 °C. The second round of PCR was qPCR and carried out in an Applied Biosystems 7500 Fast Real-Time PCR System. Reaction mixtures contained forward and reverse primer (0.5 µl of 10 µM each), 1 µl of 100% DMSO, 6 µl of nuclease-free water, 10 µl of 2× SYBR Green, and 2 µl of PCR products from the first round. The temperature profile for the reaction was: 95 °C hold for 10 min, 40 rounds of cycling (95 °C for 3 s, 60 °C for 30 s), and final melt curve program (95 °C for 15 s, 60 °C for 1 min and 95 °C for 15 s, 60 °C for 15 s). The primers (Table S1) for genes *BCL2*, *CASP3*, *CASP8*, *MLKL*, *RIPK1* and *RIPK3* were either designed or sourced from previously published studies^26–28^. Gene expression analysis was performed using the 2^−∆∆Ct^ method using the *ACTB* gene (β-*ACTIN*) as the reference gene for normalization.

### Statistical analysis

All experiments were conducted with two technical replicates and repeated three times. Two-way ANOVA was performed in R^29^ with cell type and levels of PDAs as the main factors. PDAs were not compared among each other. Pair-wise comparisons of means between different levels of PDAs were made using Tukey’s post-hoc analysis. A P value of less than 0.05 was considered statistically significant.

## Results

### Effects of PDAs on cellular metabolic activity is dose-dependent

**Figure 1:**
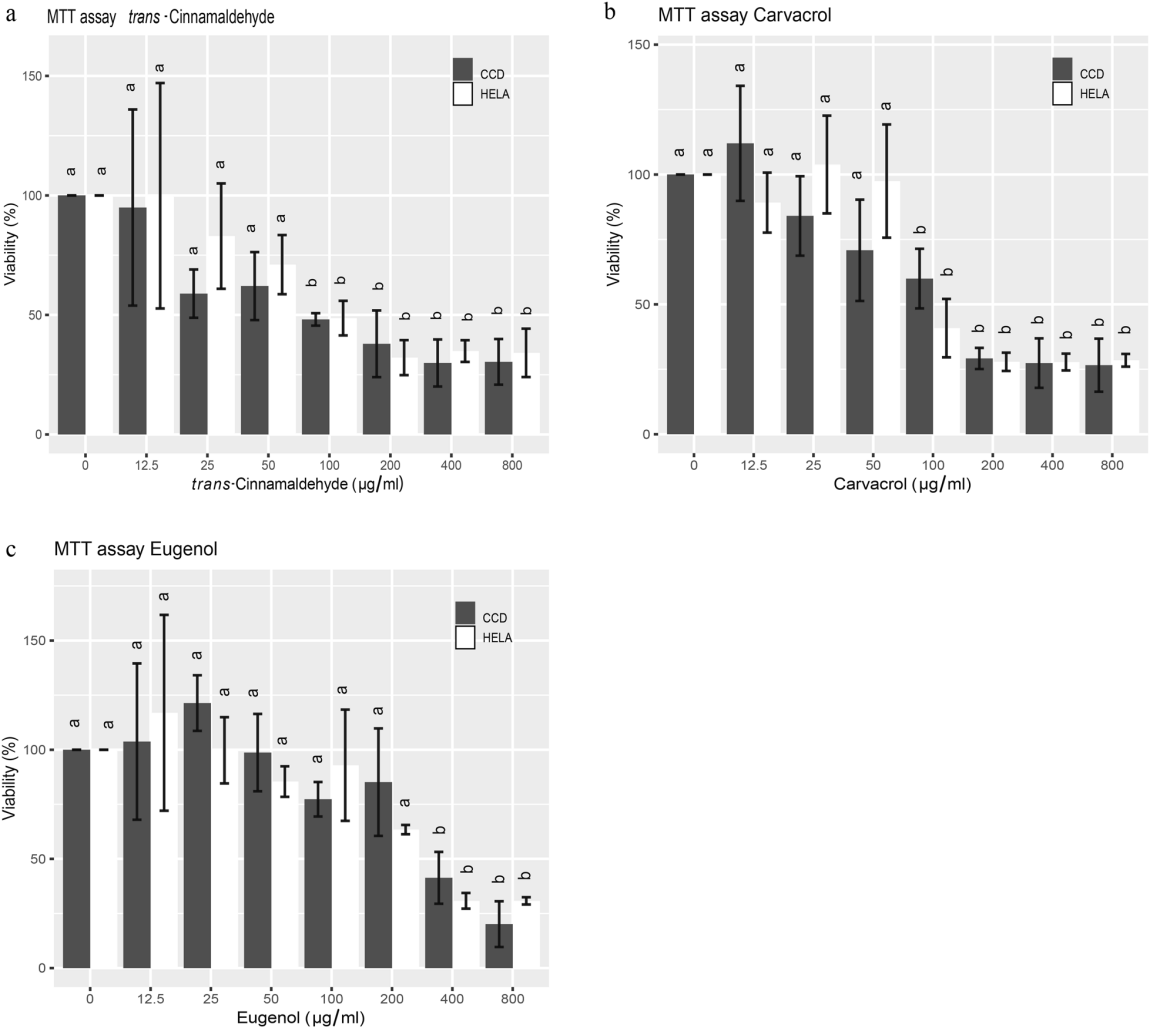
Effects of TCA (**a**), carvacrol (**b**) and eugenol (**c**) on the metabolic activity (MTT assay) of HeLa and CCD cells after 24 h of treatment. The metabolic activity of treated cells was expressed as percentages over controls without PDA treatments. Bars with different alphabet labels within cell types are significantly different (*P* ≤ 0.05).

All PDAs (TCA, EU and CAR) reduced cellular metabolism, a measure of cell viability, in a dose-dependent manner as determined by the MTT assay. Cancerous cells (HeLa) and non-cancerous fibroblasts (CCD) were equally affected by the treatments (Fig. 1). Specifically, TCA induced a gradual decrease in metabolic activity with increasing concentrations. This effect did not become significant on untreated controls until 100 µg/ml, when the metabolic activities of HeLa and CCD cells were reduced by more than half, to 48.7% and 48.13% of controls, respectively (*P* ≤ 0.05; Fig. 1a). The effect of TCA appears to level off from 200 µg/ml up to the maximum dose tested at 800 µg/ml, where metabolic activity was reduced to approximately 30% of that of untreated controls.

Similar to TCA, carvacrol also reduced metabolic activity on both cell types in a dose-dependent manner. Significant differences were observed at 100 µg/ml with viability declining to 40.8% and 59.9% of controls for HeLa and CCD cells, respectively (*P* ≤ 0.05; Fig. 1b). Finally, eugenol appeared to be less effective than TCA and carvacrol, and did not exert a significant effect on the metabolic activity up to 400 µg/ml (*P* ≤ 0.05; Fig. 1c), when metabolic activity was reduced to 30.8% and 41.34% of controls for HeLa and CCD cells, respectively.

Interestingly, while not significant, both CAR and EU increased cell viability at low levels (12.5 and 25 µg/ml). This is potentially due to the anti-inflammatory effect of these PDAs, while higher levels of PDAs became cytotoxic.

### CAR and EU induced cytotoxicity

**Figure 2:**
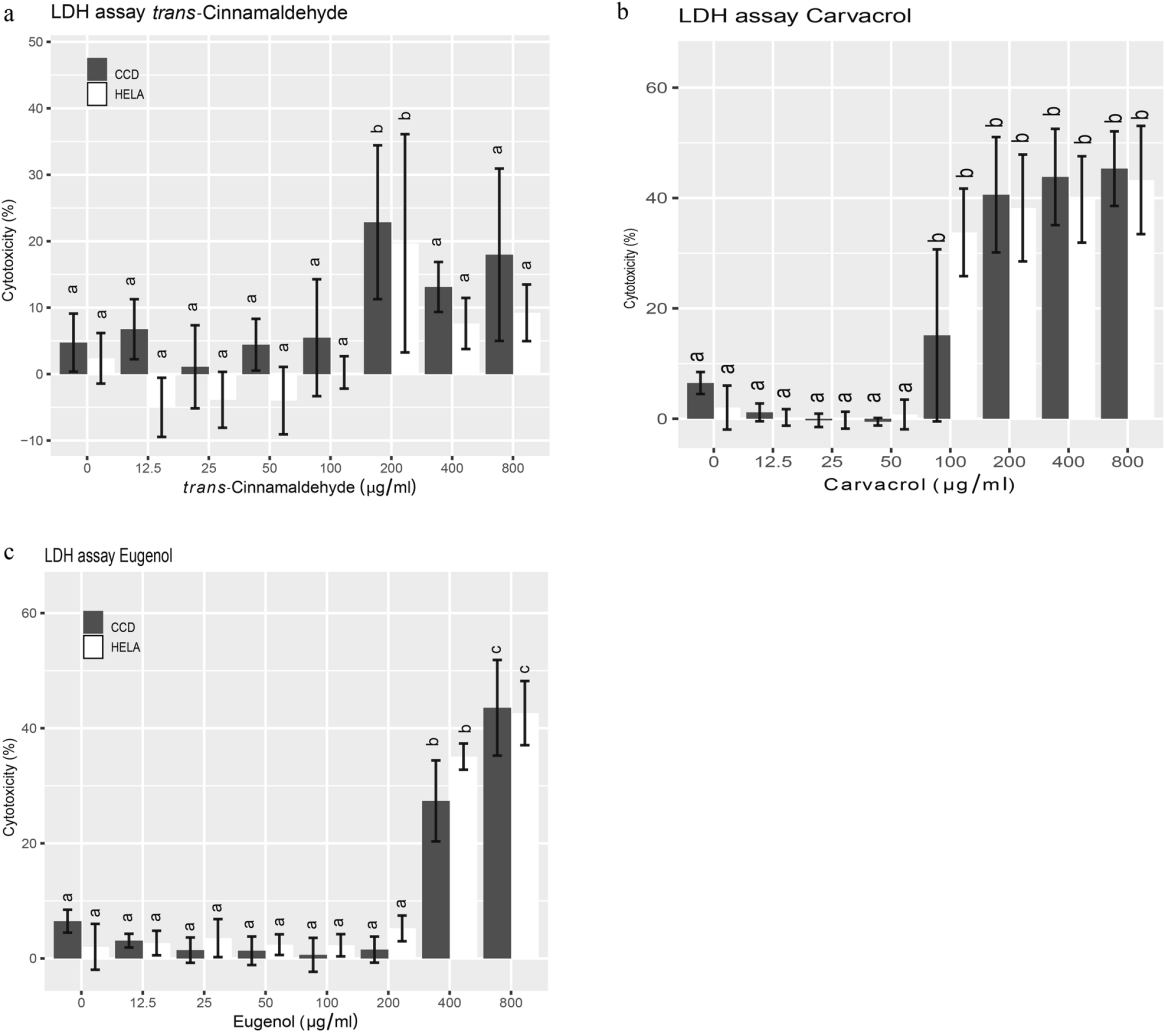
Cytotoxicity (LDH assay) of TCA (**a**), carvacrol (**b**) and eugenol (**c**) on HeLa and CCD cells after 24 h of treatment. Cytotoxicity on treated cells was expressed as percentages over un-treated controls. Bars with different alphabet labels within cell types are significantly different (*P* ≤ 0.05).

Carvacrol and eugenol, but not TCA, disrupted membrane integrity, a measure of cytotoxicity, in a dose-dependent manner as measured by LDH assay. HeLa and CCD cells were equally affected by the treatments. Specifically, carvacrol exhibited increased cytotoxicity on both cell types in a dose-dependent manner. Significant differences were observed at 100 µg/ml, with cytotoxicity increasing to 33.78% and 15.11% of untreated controls for HeLa and CCD cells, respectively (*P* ≤ 0.05; Fig. 2b). Eugenol, however, appeared to be less cytotoxic than carvacrol and did not exert a significant effect on viability until 400 µg/ml (*P* ≤ 0.05; Fig. 2c), when cytotoxicity was 35.07% and 27.37% of controls for HeLa and CCD cells, respectively. The results on carvacrol and eugenol are consistent with those from the MTT assay. Additionally, as with the MTT assay, no significant difference was observed in the response of HeLa and CCD cells to carvacrol and eugenol treatments.

Interestingly, TCA only showed significant cytotoxicity at 200 µg/ml on both cell types (*P* ≤ 0.05; Fig. 2a). Above this concentration significant differences could not be observed (400 and 800 µg/ml; Fig. 2a). This may not be surprising because TCA has been reported to interfere with LDH activity, which in turn, may have caused the large variation in the results of this assay^30^. Consequently, we believe that TCA was still exerting cytotoxicity by causing membrane damage to both cell types at all levels above 200 µg/ml, yet we did not obtain consistent readings due to the interference of TCA with the assay itself (Fig. 2a).

### Apoptosis is involved in PDA-mediated cell death

**Figure 3:**
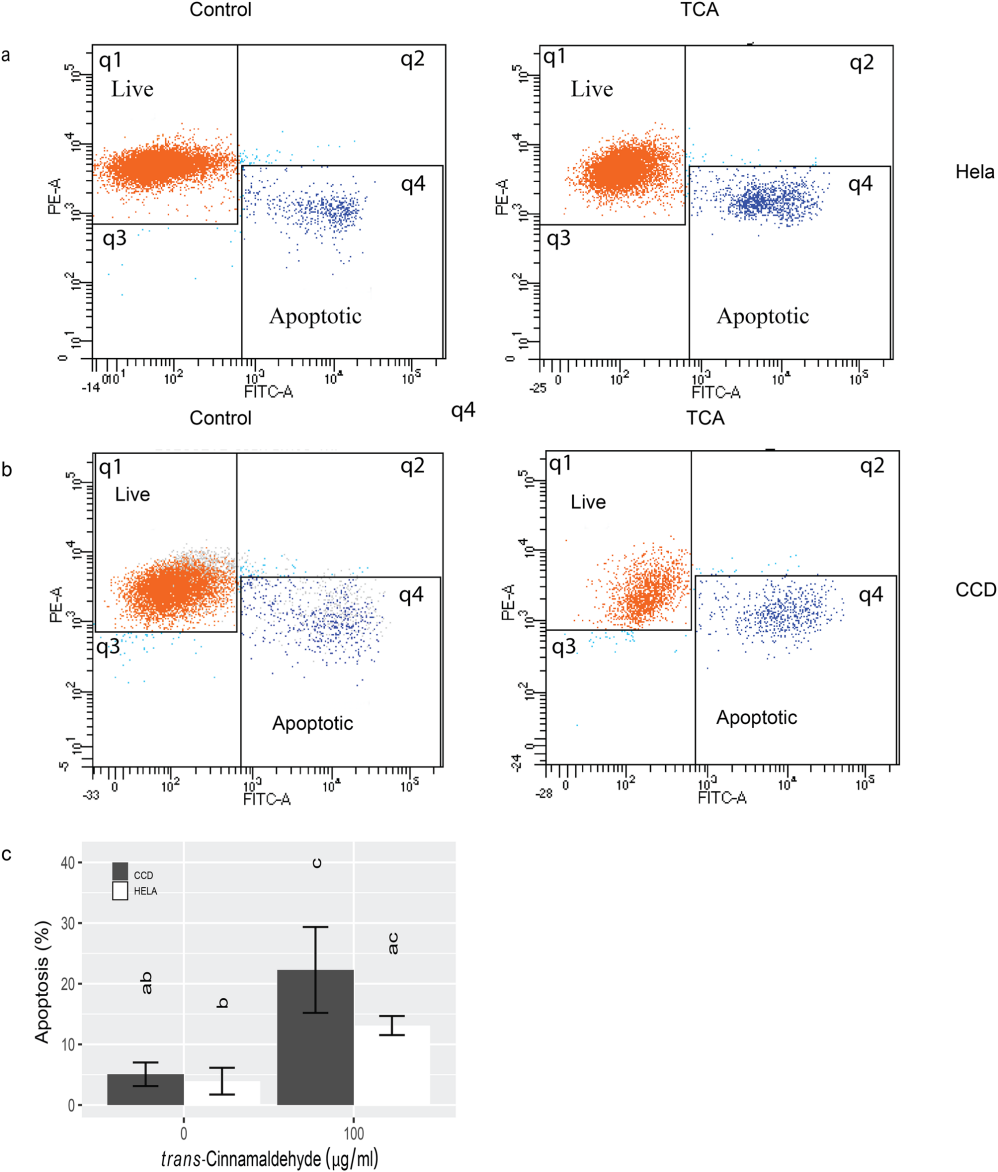
Flow cytometry analysis for apoptosis in HeLa (**a**) and CCD cells (**b**) after TCA (0 and 100 µg/ml) treatment for 4 h. In each scatterplot, live cells (red dots in q1) were stained positive for Mito tracker (red) and negative for Annexin V which indicated positive mitochondrial activity and apoptotic cells, respectively. Apoptotic cells (dark blue dots in q4) were Annexin V and Mito tracker positive. (**c**) The mean percentages of apoptotic HeLa and CCD cells before and after TCA treatment. While both cell types responded to TCA and no significant difference was found between the cell type, with CCD cells appearing to have an accelerated response.

In response to PDA treatment, both cell types rounded up, detached from the dish, and shrank in size, thereby exhibiting typical morphology of cell death (data not shown). To determine if apoptosis was involved, we quantified Annexin V staining by flow cytometry. We then selected TCA as a representative at the concentration of 100 µg/ml, the lowest level to induce significant viability change and incubated both HeLa and CCD cells for 1, 2, and 4 h (data not shown). A TCA treatment of 4 h allowed us to catch apoptosis in progress. The effects of TCA on apoptosis are shown in Fig. 3. In each scatterplot, the top left quadrant (q1) showed live cells stained red and positive for mitochondrial membrane and negative for Annexin V. The bottom right quadrant (q4) showed apoptotic or dead cells which stained negative for MitoTracker and positively for Annexin V. A basal level of apoptotic cells of approximately 5% was present in both cell types before treatments (Fig. 3c). The progression of apoptosis was captured after 4 h of TCA treatment in both cell types (Fig. 3a and b). The percentages of apoptotic cells increased to 13.1% and 22.3% for HeLa and CCD, respectively (*P* ≤ 0.05).

### Real-time PCR analysis of expression of apoptotic and necrotic genes

**Figure 4:**
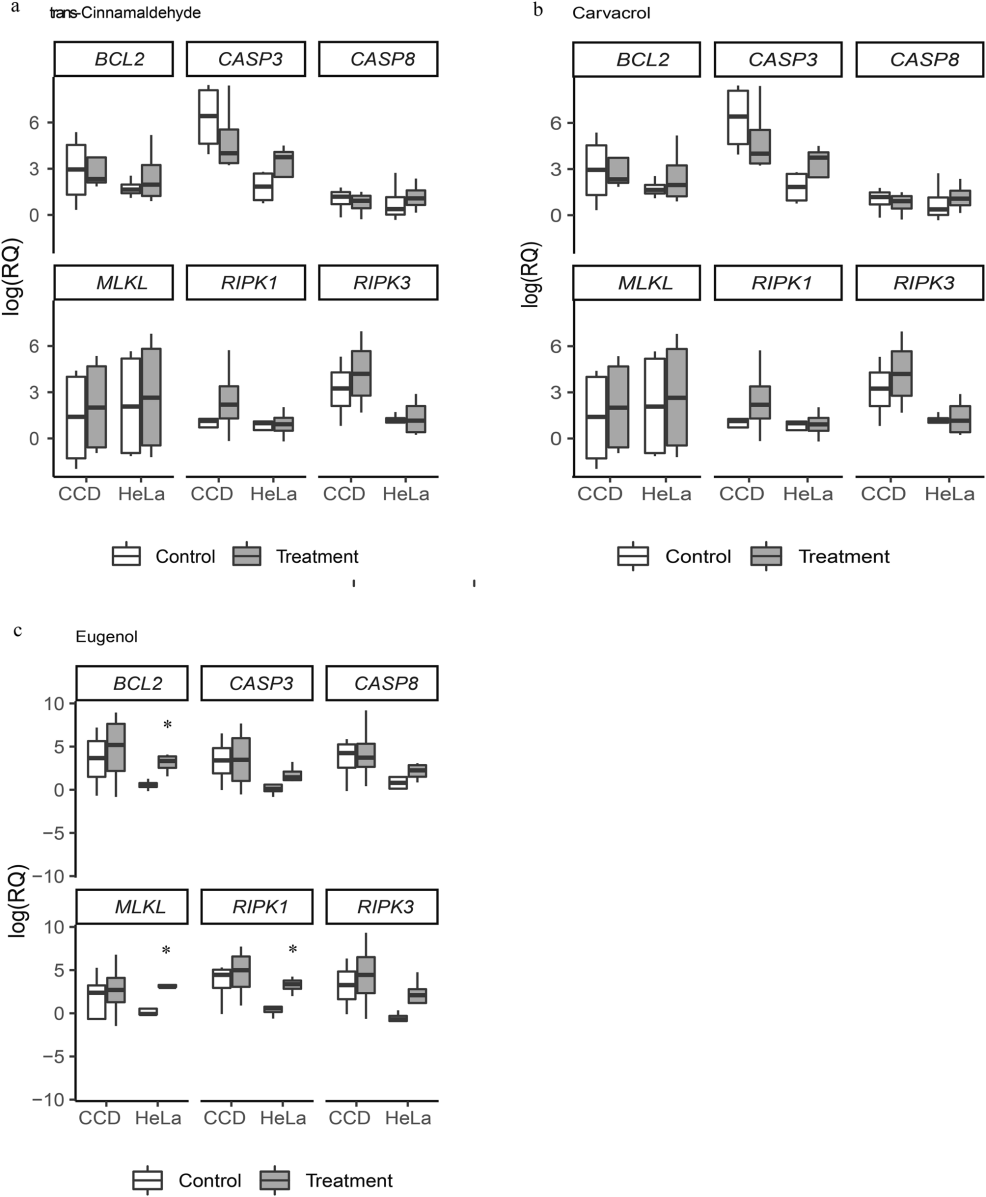
Relative levels of mRNAs for apoptotic and necrotic genes in HeLa and CCD cells treated with (**a**) TC, (**b**) CAR, and (**c**) EU. Expression levels are expressed in log (RQ, relative quantity) values. Asterisk (*) represents a significant difference between control and treated.

To determine if PDAs induced changes in the mRNA levels of apoptotic and necrotic genes, three representative genes of each pathway (*BCL2*, *CASP3*, *CASP8* for apoptosis and *MLKL*, *RIPK1*, *RIPK3* for necrosis) were chosen for analysis in treated and non-treated HeLa and CCD cells. This resulted in a total of 36 comparisons. TC and CAR treatments were not associated with significant changes in expression of genes studied between non-treated and treated cells of either type (Fig. 4a, b and Table S2). Significant changes in gene expression were only found in three comparisons. HeLa cells treated with EU showed a significant increase in mRNA levels of *BCL2, MLKL* and *RIPK1* compared to non-treated groups (*P* ≤ 0.05). CCD cells did not show any significant differences between the control and treated groups (Fig. 4c and Table S2).

## Discussion

In this study we chose one cancerous cell type, HeLa, and one non-cancerous cell line, fibroblasts (CCD), for direct comparison to determine their responses to PDA treatments. This initial examination is intended to induce additional studies in which other cancerous and non-cancerous cell lines are compared directly. Confirmation and scientific repetitions are more robust if tested in different laboratory settings and by different experimenters. We demonstrated that with increasing concentrations, TCA, carvacrol, and eugenol caused decreased cell viability and increased cytotoxicity (*P* ≤ 0.05). We also showed that apoptosis was involved in these processes.

Out of the three compounds studied, TCA and carvacrol appeared to be more effective than eugenol in reducing cell viability. TCA, however, showed different effectiveness in viability and cytotoxicity. From the large variation of the data, we believed that TCA may somehow interfere with the LDH assay. This assumption was supported by previous findings that pre-treatment of cinnamaldehyde decreased LDH activities in rats^30^.

Following PDA treatments, both cell types rounded up, detached from the dish, and shrank in size, exhibiting typical morphology of cell death. To determine if apoptosis was involved, we quantified Annexin V staining by flow cytometry which readily revealed significant apoptosis at 100 µg/ml of TCA, a concentration that significantly reduced cell viability. The percentages of apoptotic cells, ~ 20%, were much lower than those of non-viable cells (~ 70%) at the same TCA concentration in the MTT study. This was likely a result of the difference in treatment duration, 4 h vs. 24 h, between the two types of experiments. More cells would be progressively going through apoptosis as the treatment extended to 24 h.

Based on prior findings using cancer cells only including HeLa^22,23,31^, we expected the PDA treatments to affect the expression of genes in the apoptotic pathway in both cell types. No prior studies had explored the role of necrosis by PDA treatments. The only indication of necrosis was the report of a smear pattern of DNA fragmentation when human oral cancer cells were treated with eugenol^32^. We therefore aimed to determine expression of genes in apoptosis and necrosis in both cell types. We used three PDA treatments on two cell types and studied six genes. This resulted in a total of 36 comparisons. Out of these, only three comparisons were found significant, all associated with EU treatment. We believe that this may be caused by the specific doses of PDAs used in our study. Although we chose levels that produced statistical changes in cell viability and cytotoxicity, major alterations, albeit statistically insignificant due to the large variations, already occurred at much lower doses of PDAs. All three PDAs have been related to damages of cell membrane integrity, polarity, and free-radical generation in microbes^9,33–35^. We believe under the treatment regimen used in our experiments; cells were compromised due to damages to the lipid bilayer and reduced energy metabolism before they could alter gene expression. Eugenol, however, was less potent to cells, which likely were able to respond by regulating transcription. The low mRNA yield from the treated cell cultures supports the explanation of damage-related cell death. Additionally, our viability and cytotoxicity data are not compatible with gene expression data thus suggesting that gene expression alteration is not the major player under the treatment regime used.

The effects of TCA, eugenol or carvacrol on HeLa cells have been documented in six previous studies. Lee et al.^11^ found that TCA was four times more effective than eugenol in inducing cytotoxicity, a similar finding to ours. In fact, Lee et al. also found that TCA was equally effective (similar IC50) to chemotherapy drugs cisplatin and mitomycin C. Carvacrol and eugenol were found to reduce viability and/or cause cytotoxicity as well as apoptosis^22,23,31,36^. Other studies also reported potentiating effects of eugenol on a chemotherapy drug or radiation^23,31^. However, none of these prior studied included control non-cancerous cells. Our finding that PDAs affected fibroblasts and HeLa cells similarly in viability and cytotoxicity provided sufficient evidence to question whether the previously reported data on HeLa cells were specific or not.

It is also worth-noting that the concentration of PDAs previously reported to reduce cell viability and to cause cytotoxicity varied tremendously. For example, in one study, as low as 5.4 µg/ml of TCA caused a 50% change in cytotoxicity of HeLa cells^11^. In studies of eugenol, it was reported that 50 µM eugenol (approximately 8.21 µg/ml) inhibited the viability of HeLa cells by 75% with 24 h of treatment^36^. Yet in another study, it took 750 µM (approximately 123.2 µg/ml) of eugenol for 24 h to produce the same results^23^. A level between these extremes, at 350 µM (approximately 57.5 µg/ml) was also reported to have a similar effect on the viability of HeLa cells in 24h^31^. Similar to our findings, it was reported that 100 µg/ml of carvacrol reduced cell viability and increased cytotoxicity of HeLa cells to 25% and 40% of the untreated controls, respectively^19^. It is unclear why such big variations in effective PDA concentration were found against HeLa cells in previous studies. The large variations in previous published literatures should alert scientists to test the dose range in their own experimental setting. In our study, we observed large variations at the lower concentration ranges of PDAs. These lower levels sometimes produced positive effects on cell viability, most likely due to their antioxidant properties. These variations prevented us from detecting statistical significance until higher PDA levels were applied when most cells were dead upon treatments.

While searching through the literature on the anti-cancerous effects of PDAs among other cancer cell lines, we came upon only two studies that included non-cancerous cell controls. However, these studies documented conflicting results. Aydin et al. compared healthy rat neurons and N2a neuroblastoma cancer cells on their proliferative response to carvacrol treatment. Using the MTT assay they observed that both cell types were affected similarly^21^. In contrast, using human breast epithelial cells (non-cancerous) and their ras-transformed counterparts, Yan et al. reported that eugenol inhibited proliferation and intracellular ATP level of only the ras-transformed cells^37^. Cancer cells have been intensively studied for the Warburg effect of metabolism, i.e. the preferential utilization of the glycolysis pathway even in the presence of oxygen and fully functioning mitochondria^38^. More recently, however, in breast cancer cells of the mouse model, it was discovered that mitochondrial electron transport was necessary to drive de novo pyrimidine synthesis to overcome cell-cycle arrest^39^. While many cancer cells do indeed employ oxidative phosphorylation, ATP generation was found dispensable for tumorigenesis^39^. In Yan et al., eugenol was found to inhibit gene expression of the oxidative phosphorylation pathway in ras-transformed breast epithelial cells, but such inhibition was not determined on non-cancerous control cells, raising the question if eugenol can be used to target cancer cells by inhibiting the dispensable oxidative phosphorylation pathway for ATP generation^37^. Finally, while not suitable as cancer treatments by themselves, PDAs can be used in combination with chemotherapy that specifically targets cancer cells. This adjuvant effect of PDAs will strength the effectiveness of drug treatments.

## Conclusion

We conclude that although *trans*-cinnamaldehyde, carvacrol and eugenol have been reported to be anti-cancerous, they exert similar effects on a non-cancerous fibroblast cell line and cancerous HeLa cell line in regard to viability and cytotoxicity. Gene expression of the apoptosis and necrosis pathways were minimally affected under the treatment regimen.

## Supplementary Information

Supplementary Information.
