# Supplementary material for: Cytotoxic effects on cancerous and non-cancerous cells of trans-cinnamaldehyde, carvacrol, and eugenol
Source: Sci Rep. 2021 Aug 11;11:16281. doi: 10.1038/s41598-021-95394-9 (PMC8358038; doi:10.1038/s41598-021-95394-9)
Supplement: Supplementary file 1 — Supplementary Information. [file 41598_2021_95394_MOESM1_ESM.docx]

**SUPPLEMENTS**

**Table S1:** Primers used for real-time qPCR

| Genes | Primer Sequences |
| --- | --- |
| *Actin* | F 5'- CCTCGCCTTTGCCGATCC-3'  R 5'- CGCGGCGATATCATCATCC-3' |
| *BCL2* | F 5'-GATGTGATGCCTCTGCGAAG-3'  R 5'-CATGCTGATGTCTCTGGAATCT-3' |
| *CASP3* | F 5'-GCTCTGGTTTTCGGTGGGT-3'  R 5'- GAGTCCATTGATTCGCTTCCA-3' |
| *CASP8* | F 5'-AGAGTCTGTGCCCAAATCAAC-3'  R 5'-GCTGCTTCTCTCTTTGCTGAA-3' |
| *RIPK1* | F 5'-GGCATTGAAGAAAAATTTAGGC-3′  R 5′-TCACAACTGCATTTTCGTTTG-3′ |
| *RIPK3* | F 5'-GCAACATAGGAAGTGGGGCT-3′  R 5' -GGTCCCAGTTCACCTTCTCG-3′ |
| *MLKL* | F 5′-AGAGCTCCAGTGGCCATAAA-3′  R 5′-TACGCAGGATGTTGGGAGAT-3′ |

**Table S2:** P-values for the real-time qPCR results on HeLa and CCD cells. Comparisons were made using two-way ANOVA (PDAs and treatments).

|  | TC | | CAR | | EU | |
| --- | --- | --- | --- | --- | --- | --- |
|  | HeLa | CCD | HeLa | CCD | HeLa | CCD |
| *BCL2* | 0.475 | 0.745 | 0.475 | 0.745 | 0.007* | 0.691 |
| *CASP3* | 0.477 | 0.428 | 0.477 | 0.428 | 0.089 | 0.935 |
| *CASP8* | 0.664 | 0.692 | 0.664 | 0.692 | 0.090 | 0.764 |
| *MLKL* | 0.845 | 0.743 | 0.845 | 0.743 | 0.004* | 0.520 |
| *RIPK1* | 0.674 | 0.246 | 0.674 | 0.246 | 0.002* | 0.584 |
| *RIPK3* | 0.777 | 0.492 | 0.777 | 0.492 | 0.113 | 0.649 |

*=significant difference between treated and untreated within the same PDA**.**
